# Supplementary material for: Evaluating acoustic signals to reduce avian collision risk
Source: PeerJ. 2022 May 10;10:e13313. doi: 10.7717/peerj.13313 (PMC9104101; doi:10.7717/peerj.13313)
Supplement: Table S1 — The summarized qualitative flight scorings in the X- (horizontal), Y- (depth), and Z- (vertical) dimensions of the flight corridor for each bird. Within each dimension, flight descriptors are ranked from most to least common occurrence and are broken down into the percent of qualifying flights that belonged to either a control or any treatment, with color used to highlight any patterns that more strongly (green) favored a treatment or a control. [file peerj-10-13313-s002.docx]

| **Dimension** | **Rank** | **Description of flight pattern** | **% of total flights** | **% of qualifying flights from a control** | **% of qualifying flights from any treatment** |
| --- | --- | --- | --- | --- | --- |
| **X** | **1** | Gradual turn from beginning to end of flight | 38.158 | 20.690 | 79.310 |
|  | **2** | Straight for majority of flight, diverted to side near the end | 35.526 | 75.926 | 24.074 |
|  | **3 (tie)** | Diverted to side early, then continued straight | 11.184 | 41.176 | 58.824 |
|  | **3 (tie)** | Straight for the entire flight | 11.184 | 76.471 | 23.529 |
|  | **4** | Zig-zag | 3.947 | 50 | 50 |
| **Y** | **1** | Long flight (within 1 m of obstacle or past obstacle) | 48.026 | 64.384 | 35.616 |
|  | **2** | Moderate flight (within 3-1 m of obstacle at nearest distance) | 32.895 | 50 | 50 |
|  | **3** | Short flight (> 3 m from obstacle at nearest distance) | 19.079 | 13.793 | 86.207 |
| **Z** | **1** | Upward change in trajectory | 49.342 | 50.667 | 49.3333 |
|  | **2** | Consistently flew at or above speaker level | 34.211 | 59.615 | 40.385 |
|  | **3** | Consistently flew at or below speaker level | 9.868 | 33.333 | 66.667 |
|  | **4** | Downward change in trajectory | 6.579 | 20 | 80 |
